# The protective role of miR-223 in sepsis-induced mortality

**DOI:** 10.1038/s41598-020-74965-2

**Published:** 2020-10-19

**Authors:** Dan Liu, Zhiding Wang, Huijuan Wang, Feifei Ren, Yanqin Li, Sifan Zou, Jianqiao Xu, Lixin Xie

**Affiliations:** 1 College of Pulmonary and Critical Care Medicine, Chinese PLA General Hospital, 28 Fuxing Road, Beijing, 100853 China; 2 Department of Hematology and Oncology, International Cancer Center, Shenzhen University General Hospital, Shenzhen University Health Science Center, Shenzhen, 518000 China

**Keywords:** Immunology, Diseases, Risk factors

## Abstract

Lymphocyte apoptosis appears to play an important role in immunodysfunction in sepsis. We investigated the role of miR-223 in cell proliferation and apoptosis to identify potential target downstream proteins in sepsis. We recruited 143 patients with sepsis and 44 healthy controls from the Chinese PLA General Hospital. Flow cytometry was used to sort monocytes, lymphocytes, and neutrophils from fresh peripheral blood. A miR-223 mimic and inhibitor were used for transient transfection of Jurkat T cells. Quantitative reverse transcriptase-polymerase chain reaction (qRT-PCR) was used to assess expression of the miRNAs in cells. Western blot analysis was performed to measure protein expression. We evaluated the cell cycle and apoptosis by using flow cytometry (FCM) and terminal deoxynucleotidyl transferase dUTP nick end labeling (TUNEL). Expression of miR-223 was significantly higher in the survivor group than in the nonsurvivor group. Multiple linear regression analysis revealed that SOFA scores correlated negatively with miR-223 and monocyte counts, with β coefficients (95% CI) of − 0.048 (− 0.077, − 0.019) and − 47.707 (− 83.871, − 11.543), respectively. miR-223 expression also correlated negatively with the percentage of apoptosis in lymphocytes. The rate of apoptosis in the miR-223 mimic group was significantly lower than that of the negative control, with an adverse outcome observed in the miR-223 inhibitor group. We also found that miR-223 enhanced the proliferation of Jurkat T cells and that inhibiting miR-223 had an inhibitory effect on the G1/S transition. We conclude that miR-223 can serve as a protective factor in sepsis by reducing apoptosis and enhancing cell proliferation in lymphocytes by interacting with FOXO1. Potential downstream molecules are HSP60, HSP70, and HTRA.

## Introduction

Sepsis is a life-threatening condition caused by multiple infections^1^. Despite advances in antibiotic therapy and modern life support, the fatality rate of patients with sepsis is as high as 30–60% worldwide^2^. Early sepsis is characterized by an excessive inflammation syndrome called the “cytokine storm.” Many patients may survive the initial hyperinflammatory phase of sepsis but die in an immunosuppressive state^3^. In this state, both innate and adaptive immunodysfunction occur, resulting in ineffective clearance of septic foci, increased vulnerability to secondary infections, and reactivation of latent infections^4^. As such, the phenomenon of sepsis-induced immunosuppression appears to play an important role in sepsis-induced morbidity and mortality. Several mechanisms may play a role in the pathophysiology of sepsis-induced immunosuppression. Apoptosis of immune cells is a significant mechanism of immunosuppression because it inhibits the ability of these cells to eradicate infection. The underlying mechanisms include increased apoptosis of lymphocytes, decreased antigen-presenting capacity of monocytes, and disordered apoptosis of neutrophils^5^. Proapoptotic factors (such as soluble-fas, fas ligand), which may activate caspases^6^, have been shown to reflect immune cell apoptosis and indicate immunoparalysis in sepsis^7,8^.

MicroRNAs (miRNAs) are endogenous noncoding small RNAs^9^ that have been shown to widely participate in sepsis. Indeed, evidence has shown that miRNAs participate in immunosuppression by influencing immune cell polarization and expression of proinflammatory cytokines. Van et al. showed that overexpression of miR-31 in stimulated human CD4 T cells promotes a proinflammatory phenotype with increased levels of interferon-γ (INF-γ) and reduces expression of interleukin (IL)-2 and IL-4. In contrast, transfection of anti-miR-31 directs cells toward a TH2 phenotype^10^.

Delays in treatment are associated with a worse prognosis^11^, which highlights the importance of a timely diagnosis to reduce the morbidity and mortality of sepsis patients. Biomarkers, which have been widely investigated in recent years, might contribute to the prompt identification of sepsis. Our meta-analysis based on these studies also highlighted the clinical use of these biomarkers^12,13^. However, further basic biology research based on these biomarkers is warranted. In our previous study, we used Solexa sequencing to show that serum miR-223 is significantly differentially expressed between survivors and nonsurvivors of sepsis^14^; using TargetScan, we also found that forkhead boxO1 (FOXO1) is predicted to be a target gene of miR-223^15^. Accumulation of FOXO1 in the nucleus activates downstream apoptosis genes (such as FasL and Trail)^16^. Previous studies have also shown that unphosphorylated FOXO1 is active and can contribute to apoptosis and cell cycle dysfunction^17^. Therefore, we aimed to address the potential role of miR-223 in apoptosis by regulating FOXO1 in sepsis.

A recent study showed that miR-223 was overexpressed after splenectomy, inducing an immunosuppressive state and suggesting that miR-223 may regulate immune changes^18^. miR-223 has also been widely investigated as a biomarker for early diagnosis and a therapeutic target against inflammatory diseases^19^. Nonetheless, no research has investigated how miR-223 regulates immunity in sepsis. Based on our previous findings^14,15^, we evaluated the potential regulatory mechanism of miR-223 in apoptosis related to sepsis and further explored the molecular mechanisms underlying the miR-223 regulation of apoptosis in vitro. To the best of our knowledge, this is the first study to directly address the potential role of miR-223 in immune cell apoptosis and to determine the mechanism of immunosuppression in sepsis. Our report provides a novel target for the development of a therapy for sepsis.

## Methods

### Study population

This study was approved by the Committee on Ethics of the Chinese PLA General Hospital and followed the Helsinki guidelines. All of the patients or their relatives agreed to participate in the research and signed an informed consent form. Informed consent was also obtained from all healthy controls involved.

Patients were recruited from the Respiratory Intensive Care Unit (RICU), the Emergency Intensive Care Unit (EICU), or the Department of Surgery’s ICU of the Chinese PLA General Hospital from July 2014 to April 2017. Blood samples were collected within 24 h after sepsis diagnosis. All patients with sepsis met the international guidelines for the management of sepsis and septic shock developed in 2016^20,21^.

### RNA isolation and real-time PCR

Peripheral arterial blood from patients with sepsis was collected within 24 h after their diagnosis. All samples were anticoagulated with ethylenediaminetetraacetic acid. Whole blood was treated with erythrocyte lysis buffer. For each 200-μL sample of blood, 1 ml TRIZOL (Tiangen Biotech Company, Beijing, China) was added and stored at − 80 °C. Blood cells from fresh peripheral blood were sorted into monocytes, lymphocytes, and neutrophils using flow cytometry (FCM). Total RNA was extracted from 200 μL of the blood sample, which had been stored at − 80 °C, as described above. miRNA levels in the total RNA were detected by real-time quantitative PCR (qRT-PCR) using a miRcute miRNA first-strand cDNA synthesis kit and miRcute miRNA qPCR detection kit (SYBR) (Tiangen Biotech Company). RNA levels were detected using a FastQuant cDNA synthesis kit and a SuperReal qRT-PCR detection kit (Tiangen Biotech Company). All procedures followed the manufacturer’s instructions. qRT-PCR was performed in triplicate. To normalize the expression levels of miRNAs, we used 5S rRNA as an internal control. The threshold cycle (CT) is defined as the fractional cycle number at which the fluorescence passes the fixed threshold.

### Western blotting

Human T-cell leukemia Jurkat cells were grown in RPMI-1640 medium supplemented with 10% heat-inactivated FBS (Sigma, USA) and 2 mM l-glutamine under an atmosphere of 95% air and 5% CO_2_ at 37 °C. A miR-223 mimic, inhibitor, and negative control (Thermo Scientific, USA) were transiently transfected into these cells using Lipofectamine 2000 Transfection Reagent (Invitrogen, USA). The miR-223 mimic, inhibitor, and negative control (40–80 nM) were added to 6-well plates at a concentration of 2 × 105/2 ml/well on the day of transfection, and effects on mature miRNAs in the cells were assayed 48 h later. An anti-Fas antibody (CH-11, MBL, Nagoya, Japan) was used to induction apoptosis in Jurkat cells. CH-11 (20 µg/ml) was added to the medium at a concentration of 4–6 × 10^5^/ml cells at 48–72 h after transfection.

Protein samples were collected and examined by Western blotting. Antibodies, including anti-FOXO1 (C29H4) rabbit mAb (Cell Signaling Technology, USA), anti-ACVR2A (Cell Signaling Technology), and anti-GAPDH (14C10) rabbit mAb (Cell Signaling Technology), were used at a 1:1000 dilution. Alkaline phosphatase-conjugated goat anti-rabbit IgG or anti-mouse IgG antibodies (KPL, CA, USA) were used at a 1:20,000 dilution. Human Apoptosis Antibody Array-Membrane (Abcam, ab134001) was used following the manufacturer’s protocol, and targets included Bad, Bax, Bcl-2, Bcl-w, BID, BIM, Caspase3, Caspase8, CD40, CD40 L, cIAP-2, cytoC, DR6, Fas, FasL, HSP27, HSP60, HSP70, HTRA, IGF-I, IGF-II, IGFBP-1, IGFBP-2, IGFBP-3, IGFBP-4, IGFBP-5, IGFBP-6, IGF-1 sR, livin, p21, p27, p53, SMAC, Survivin, sTNF-R1, sTNFR2, TNF-alpha, TNF-beta, TRAILR-1, TRAILR-2, TRAILR-3, TRAILR-4, and XIAP.

### Assessment of apoptosis and proliferation

Transfected cells were induced to undergo apoptosis after 48–72 h by adding an anti-Fas/CD95 (CH-11) antibody. After 4 h of apoptosis induction, the cells were harvested and stained with a FITC Annexin V Apoptosis Detection Kit (BD Pharmingen, USA). Then, each group was tested for apoptosis by FCM. The cells were harvested and fixed with 4% paraformaldehyde. Apoptosis analysis was performed using a TUNEL staining kit (Abcam, USA). All fluorescent images were obtained using a Leica DM3000 microscope equipped with a DFC 420 camera (Leica, Germany). Cells were also harvested and fixed with 4% paraformaldehyde. Apoptosis was also examined using a terminal deoxynucleotidyl transferase dUTP nick end labeling (TUNEL) assay with a TUNEL staining kit (Abcam, USA). As above, fluorescent images were obtained with a Leica DM3000 and a DFC 420 camera (Leica).

Cell proliferation was determined using a WST-1 assay kit (Beyotime, China) according to the manufacturer’s instructions. Briefly, cells were seeded in triplicate in complete RPMI 1640 medium in 96-well plates (2000 cells/well in 100 μl culture medium) and incubated overnight. A total of 10 μl WST-1 was added and incubated for another 2 h at 37 °C.

To synchronize the cell cycle, RPMI-1640 medium without FBS was used 24 h before transfection. The miR-223 mimic, inhibitor, and negative control were transfected into the cells, as mentioned above, which were then washed in PBS (pH 7.4) and fixed for 30 min in ice-cold 70% ethanol. The fixed cells were washed three times in PBS (pH 7.4) and incubated with RNase A (0.02 mg/ml) for 30 min at 37 °C. Nuclei were stained with propidium iodide (40 μg/ml) and analyzed by flow cytometry.

### Statistical analysis

The expression levels of miRNAs were calculated using 2^−∆CT^, where ∆Ct = Ct miRNA − Ct 5SsnRNA. The results for normally distributed variables are given as the means ± standard deviations. The results for the variables not normally distributed are summarized as medians; the Mann–Whitney U-test was applied for comparisons. Pearson’s correlation analysis was used to evaluate the relationship between two variables. Differences were statistically evaluated by one-way ANOVA followed by Fisher’s PLSD. *P* values < 0.05 were considered significant. Error bars are used to indicate the SD. All statistical analyses were performed using SPSS 20.0 (IBM, New York, USA).

## Results

### General clinical characteristics of sepsis and healthy control groups

A total of 143 patients with sepsis and 44 healthy controls were included in this study. The 28-day mortality of the sepsis patients was 22.4%. These sepsis patients and controls were matched by age (*P* = 0.922) and sex (*P* = 0.129). The characteristics of the study population are shown in Table 1.

**Table 1:** Clinical characteristics of the 143 sepsis patients.

Category	Variables	Sepsis patients(n = 143)
Demographic parameters	Gender(n)(male/female)	76/67
	Age(years)	61.03 ± 18.375
Clinical parameters	APACHEII score	15.08 ± 8.38
	SOFA score	5.97 ± 4.906
	CRP(mg/dl)	12.410 ± 45.166
	PCT(ng/ml)	4.658 ± 16.112
	WBC(× 109/l)	12.602 ± 6.007
	miR-223(cycles)	21.195 ± 29.703
	28-day mosrtality(%)	22.378
Co-morbid conditions	CHD(n)	22
	Cancer(n)	10
	Hypertention(n)	28
	Diabetes(n)	20
	COPD(n)	18
ICU type	Medical(n)	51(35.664%)
	Surgical(n)	44(30.769%)
	Emergency(n)	48(33.566%)
Source of infection	Lung(n)	65(45.455%)
	Abdomen(n)	30(20.979%)
	Postsurgery(n)	28(19.580%)
	Catheter/blood stream(n)	4(2.797%)
	Other(n)	16(11.189%)

APACHE II score, SOFA score, CRP, PCT, WBC, miR-223 are all given as median (range).

*CHD* coronary heart disease; *COPD* chronic obstructive pulmonary disease; *WBC* white blood cell. *APACHE* Acute Physiology and Chronic Health Evaluation. *CRP* C-reactive protein. *PCT* procalcitonin. *SOFA* Sequential Organ Failure Assessment.

### Expression of miR-223 in white blood cells in sepsis

**Figure 1:**
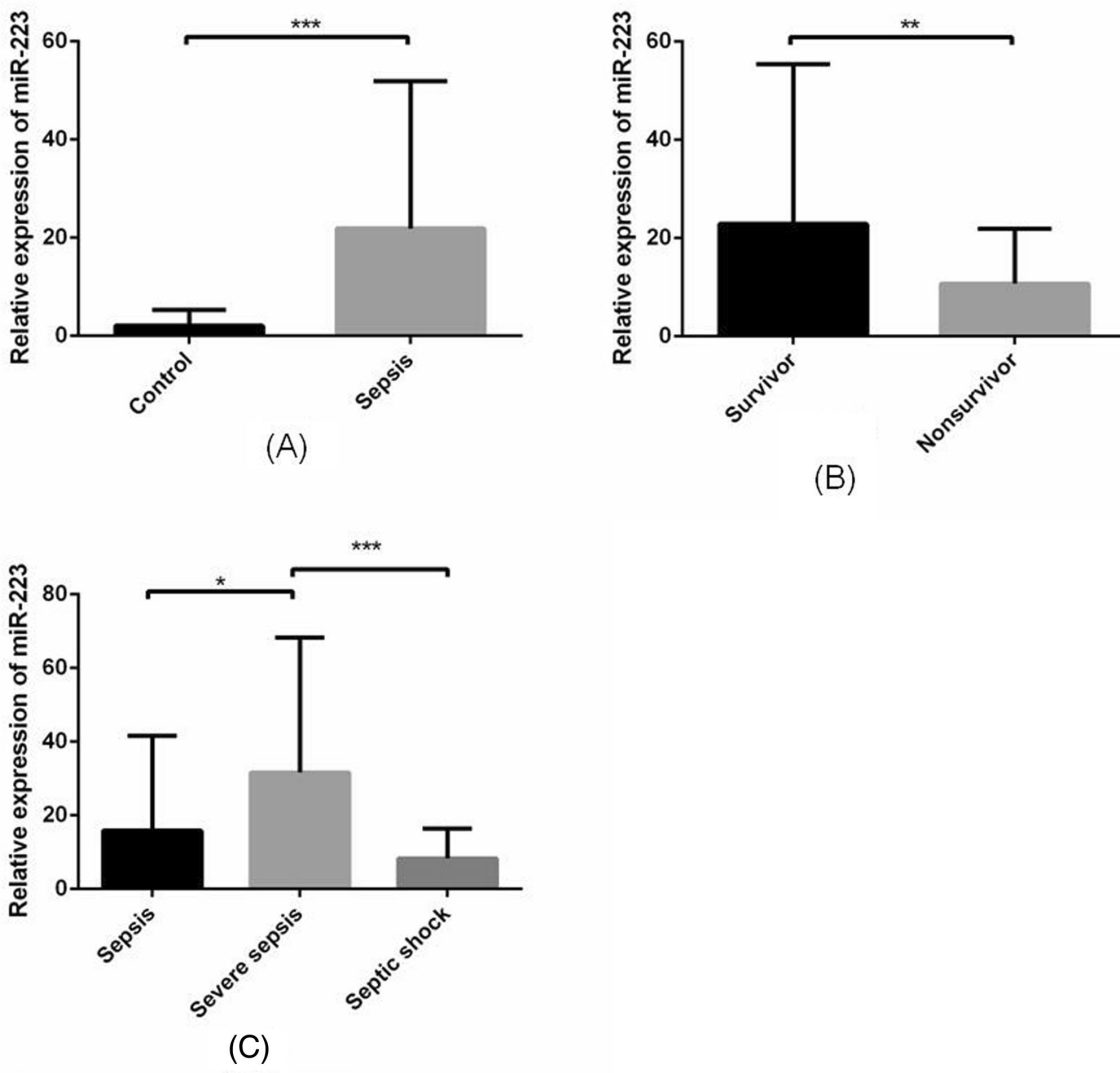
Expression of miR-223 in white blood cells. (**A**) miR-223 was significantly increased in the sepsis group compared with the controls. (**B**) miR-223 was significantly decreased in the nonsurvivor group compared with the survivor group (*P* = 0.001). (**C**) miR-223 increased significantly more in the severe sepsis group than in the sepsis group (*P* = 0.011) and decreased significantly less in the septic shock group than in the severe sepsis group (*P* < 0.001). *P < 0.05, **P < 0.01, ***P < 0.001.

Compared with healthy controls, miR-223 increased significantly in sepsis patients (*P* < 0.001) (Fig. 1A). We further divided the sepsis group into a survivor group and a nonsurvivor group according to 28-day mortality and found that expression of miR-223 was significantly higher in the survivor group than in the nonsurvivor group (*P* = 0.001) (Fig. 1B).

We used binary logistic regression to evaluate any possible associations between miR-223 and the risk of death. Expression of miR-223 along with sex, age, lymphocyte counts, monocyte counts, white blood cell (WBC) counts, PCT, CRP, IL-6, D-dimer, Acute Physiology and Chronic Health Evaluation II (APACHE II) scores, and Sepsis-Related Organ Failure Assessment (SOFA) scores were included in univariate logistic regression analysis. Sex, miR-223, APACHE II scores, and SOFA scores showed statistically significant differences of *P* < 0.05. These variables were entered into binary logistic regression analysis, which showed that APACHE II scores were independent risk factors for mortality in sepsis patients (Table 2).

**Table 2:** Univariate and binary logistic regression analysis of miR-223 between survivors and nonsurvivors.

Variables	Univariate		Multivariate	
	OR(CI)	*P*	OR(CI)	*P*
Sex	1.041 (1.014–1.068)	0.003	0.742 (0.249–2.210)	0.742
Age	1.100 (0.472–2.561)	0.825	–	–
Lymphocyte counts	0.271 (0.000–263.417)	0.71	–	–
Monocyte counts	0.000 (0.000–2.120)	0.061	–	–
WBC counts	0.981 (0.916–1.051)	0.587	–	–
PCT	0.975 (0.927–1.024)	0.311	–	–
CRP	0.994 (0.962–1.026)	0.694	–	–
IL-6	0.998 (0.994–1.002)	0.314	–	–
D-Dimer	0.987 (0.910–1.070)	0.749	–	–
APACHEII	1.307 (1.180–1.449)	< 0.001	1.270 (1.140–1.415)	< 0.001
SOFA score	1.265 (1.142–1.401)	< 0.001	1.100 (0.960–1.260)	0.168

*OR* odds ratio; *CI* confidence interval.

SOFA scores are widely used to evaluate the severity of sepsis. Multiple linear regression analysis was employed to evaluate the relationship between SOFA scores and confounding factors that may be related to the severity of sepsis, including miR-223, age, lymphocyte counts, monocyte counts, white blood cell (WBC) counts, PCT, CRP, IL-6, and D-dimer. In univariate models, a significant correlation was found between SOFA scores and miR-223 (correlation coefficient = − 0.32, *P* = 0.001), D-dimer (correlation coefficient = 0.361, *P* < 0.001), monocyte counts (correlation coefficient = − 0.246, *P* = 0.007), and IL-6 (correlation coefficient = 0.175, *P* = 0.042). Moreover, multiple linear regression analysis revealed that SOFA scores correlated significantly with miR-223, monocyte counts, and D-dimer, with β coefficients (95% CI) of − 0.048 (− 0.077, − 0.019), − 47.707 (− 83.871, − 11.543), and 0.415 (0.192, 0.637), respectively. miR-223 and monocyte counts correlated negatively with SOFA scores, but D-dimer correlated positively (Table 3).

**Table 3:** Influencing factors on SOFA scores by multiple linear regression analysis.

Confounding factors	Pearson correlation coefficient	*P* value	β Coefficient (95% CI)
MiR-223	− 0.32	0.001	− 0.048 (− 0.077, − 0.019)
D-Dimer	0.361	*P* < 0.001	0.415 (0.192, 0.637)
Age	0.055	0.294	
Monocyte counts	− 0.246	0.007	− 47.707 (− 83.871, − 11.543)
Lymphocyte counts	0.072	0.24	
WBC counts	0.032	0.378	
CRP	0.11	0.139	
PCT	0.111	0.138	
IL-6	0.175	0.042	

### Expression of miR-223 in monocytes, lymphocytes and neutrophils

We further assessed expression of miR-223 in various types of blood cells. Fresh peripheral blood from 12 sepsis patients was sorted into monocytes, lymphocytes, and neutrophils by flow cytometry. Monocytes, lymphocytes, and neutrophils were collected, and qRT-PCR was used to detect expression of miR-223 in these cells. The characteristics and hematological parameters of the 12 sepsis patients are shown in Table S3. These 12 patients were subsequently grouped into survivors and nonsurvivors based on 28-day mortality. miR-223 levels were significantly higher in lymphocytes from survivors than in those from nonsurvivors (Fig. S1A,B). Thus, we investigated the regulatory function of miR-223 in lymphocytes from sepsis patients.

### miR-223 correlates negatively with early apoptosis of lymphocytes

**Figure 2:**
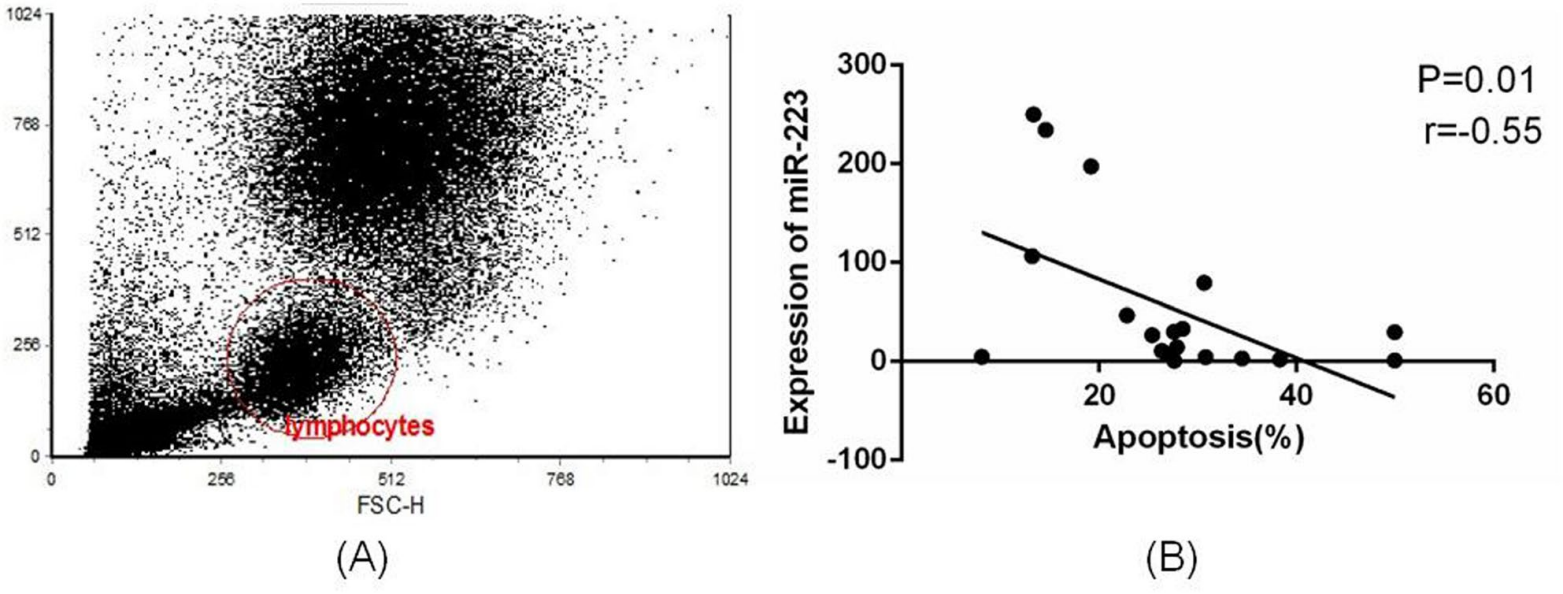
miR-223 correlates negatively with lymphocyte apoptosis. (**A**) Flow cytometry was used to separate lymphocytes and detect the proportions of apoptosis. (**B**) Lymphocytes were collected by using flow cytometry, and qRT-PCR was used to detect expression of miR-223 in these cells. Expression of miR-223 correlated negatively with lymphocyte apoptosis.

Next, we investigated the relationship between miR-223 and lymphocyte apoptosis in sepsis. Lymphocytes were collected by flow cytometry, and qRT-PCR was used to detect expression of miR-223. The percentage of lymphocyte apoptosis was measured by FCM in the same blood sample (Fig. 2A), and we found that expression of miR-223 correlated negatively with the percentage of apoptosis in lymphocytes (Fig. 2B).

### Role of miR-223 expression in apoptosis and proliferation

miR-223 mimic and inhibitor were used to establish miR-223-overexpressing and -knockout transiently transfected cell lines, respectively, and we assessed the efficacy of transfection. The results showed that miR-223 was overexpressed in Jurkat T cell lines in the mimic group and reduced in the inhibitor group compared to the NC group (Fig. S2).

**Figure 3:**
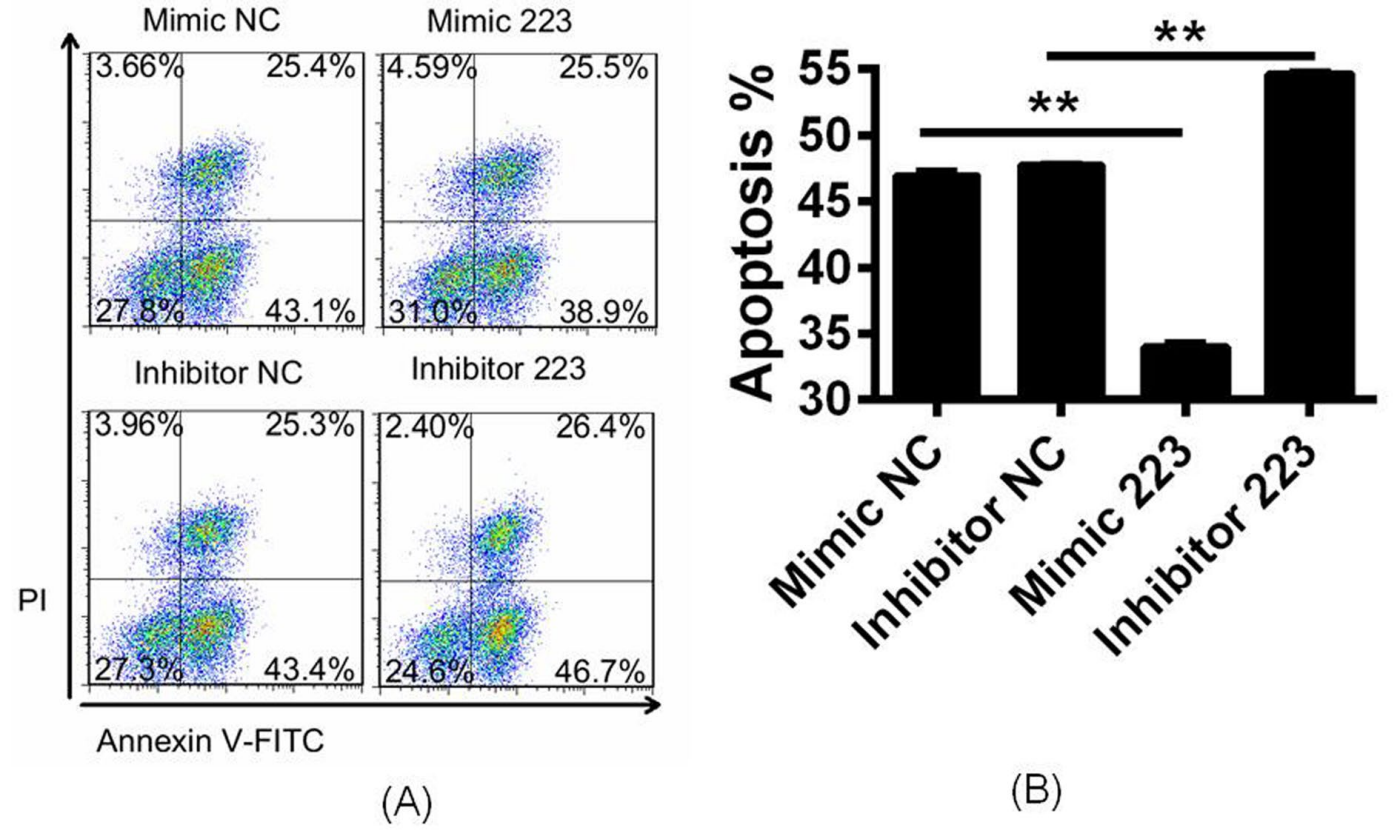
miR-223 prevents Fas-mediated apoptosis. (**A**) Apoptosis was measured in Jurkat T cells using annexin V labeling. (**B**) The rate of apoptosis in the miR-223 mimic group was significantly lower than that of the negative control, but that the miR-223 mimic group was significantly higher than that of the control, indicating that miR-223 prevents Fas-mediated apoptosis.

Next, CH-11 was used for to induce apoptosis in each group using flow cytometry. The apoptosis rate in the miR-223 mimic group was significantly lower than that in the negative control; however, apoptosis was significantly higher in the miR-223 inhibitor group than in the control (Fig. 3A,B). TUNEL assays were used to observe the percentage of apoptosis, whereby TUNEL-positive nuclei due to DNA fragmentation were observed as brown condensed spots. Compared with the NC group, there was a significant increase in apoptosis in the miR-223 mimic group and a significant decrease in the miR-223 inhibitor group after cells were treated with CH-11 (Fig. S3). These results indicate that miR-223 prevented Fas-mediated apoptosis.

In the WST-1 assay, a larger OD value indicated more active cell proliferation. The OD value of the miR-223 mimic group was significantly higher than that of the control, whereas the inhibitor group showed the reverse trend. The results indicate enhanced proliferation of Jurkat T cells in the miR-223 mimic group. Conversely, inhibition of miR-223 decreased the proliferation of Jurkat T cells after CH-11 treatment (Fig. S4).

### Inhibiting miR-223 suppresses the G1/S transition

**Figure 4:**
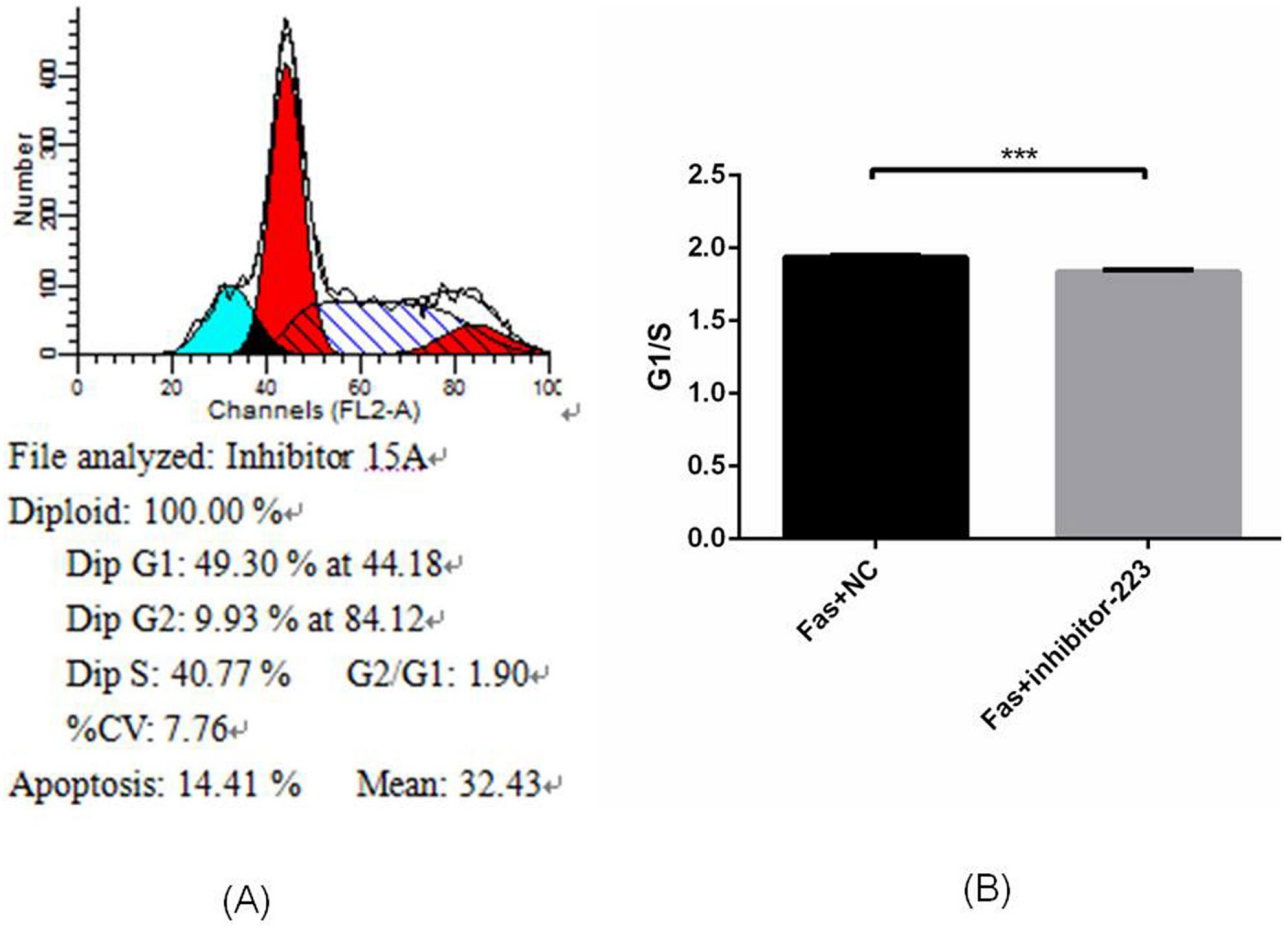
Inhibiting miR-223 has an inhibitory effect on the G1/S transition. (**A**) The cell cycle was analyzed by flow cytometry. (**B**) G1/S-phase cells were significantly reduced in the miR-223 inhibitor group, which indicated that the cell cycle was disturbed when miR-223 was inhibited.

The cell cycle was analyzed by flow cytometry (Fig. 4A). According to the results, G1/S-phase cells were significantly reduced in the miR-223 inhibitor group (Fig. 4B), indicating that the cell cycle was disturbed when miR-223 was inhibited.

### Potential target genes of miR-223

**Figure 5:**
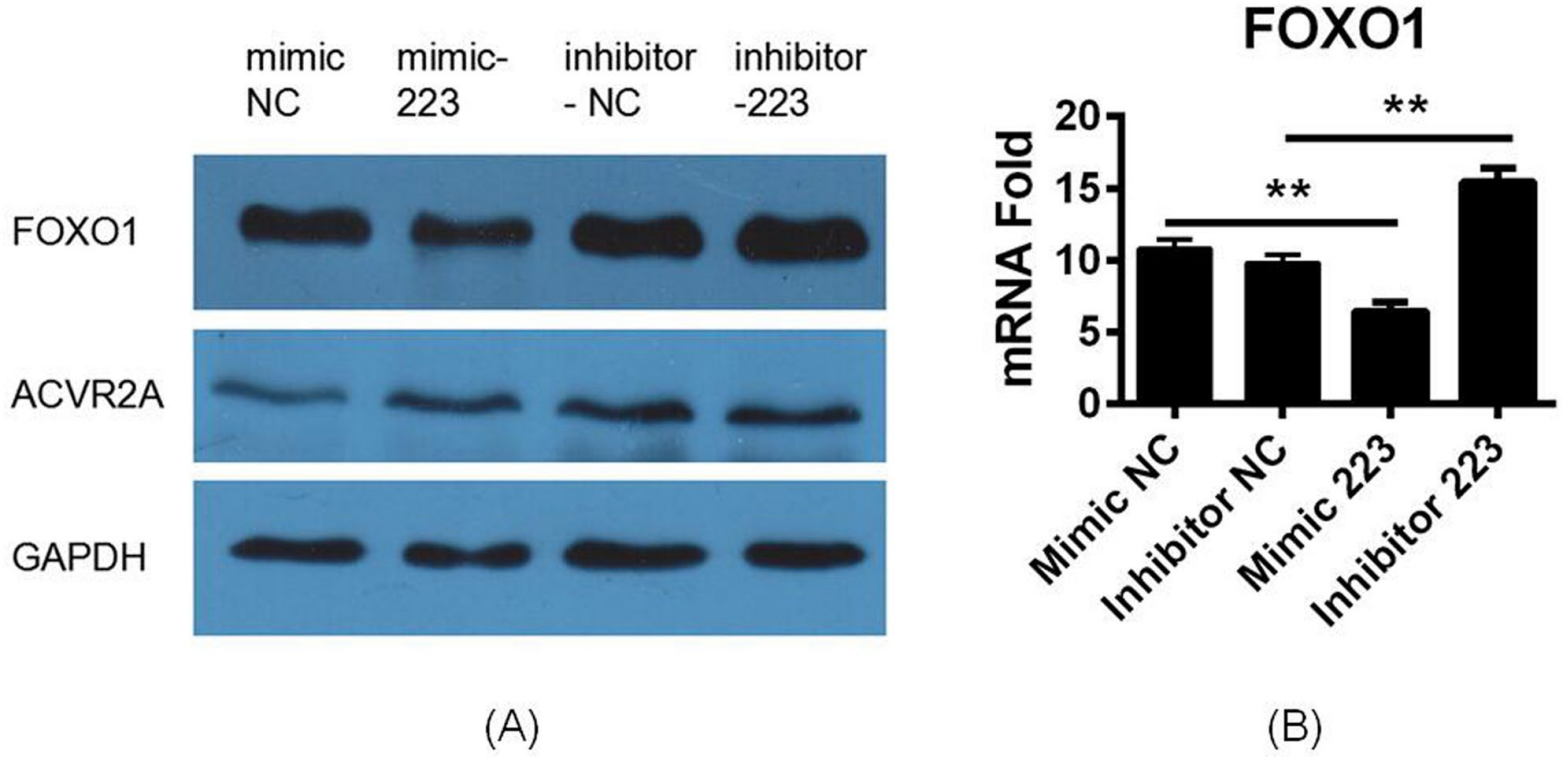
miR-223 affects apoptosis by regulating FOXO1. (**A**) In the miR-223 mimic group, protein expression of FOXO1 was significantly decreased compared with the controls, whereas the inhibitor group showed the reverse trend. ACVR2A was not significantly changed in either group. (**B**) mRNA expression of FOXO1 was significantly decreased in the mimic group and increased in the inhibitor group compared with the control.

In our earlier research^15^, ACVR2A (activin A receptor, type IIA), FOXO1, and SLC4A4 (solute carrier family 4, member 4) were predicted to be common target genes of miR-223. According to the KEGG pathway database, SLC4A4 is only involved in pancreatic and bile secretion. Therefore, we selected FOXO1 and ACVR2A for further target gene identification. In the miR-223 mimic group, FOXO1 protein expression was significantly decreased compared with the controls, whereas the inhibitor group showed the reverse trend. ACVR2A exhibited very low expression and was not significantly changed in either group (Fig. 5A). Supplemental files provide the original figure of Fig. 5 (Fig. S5); the data in Fig. 5 were quantified using ImageJ (Table S1). mRNA expression of FOXO1 was significantly decreased in the mimic group and increased in the inhibitor group compared with the control (Fig. 5B). These results indicate that FOXO1 is a potential target gene of miR-223.

**Figure 6:**
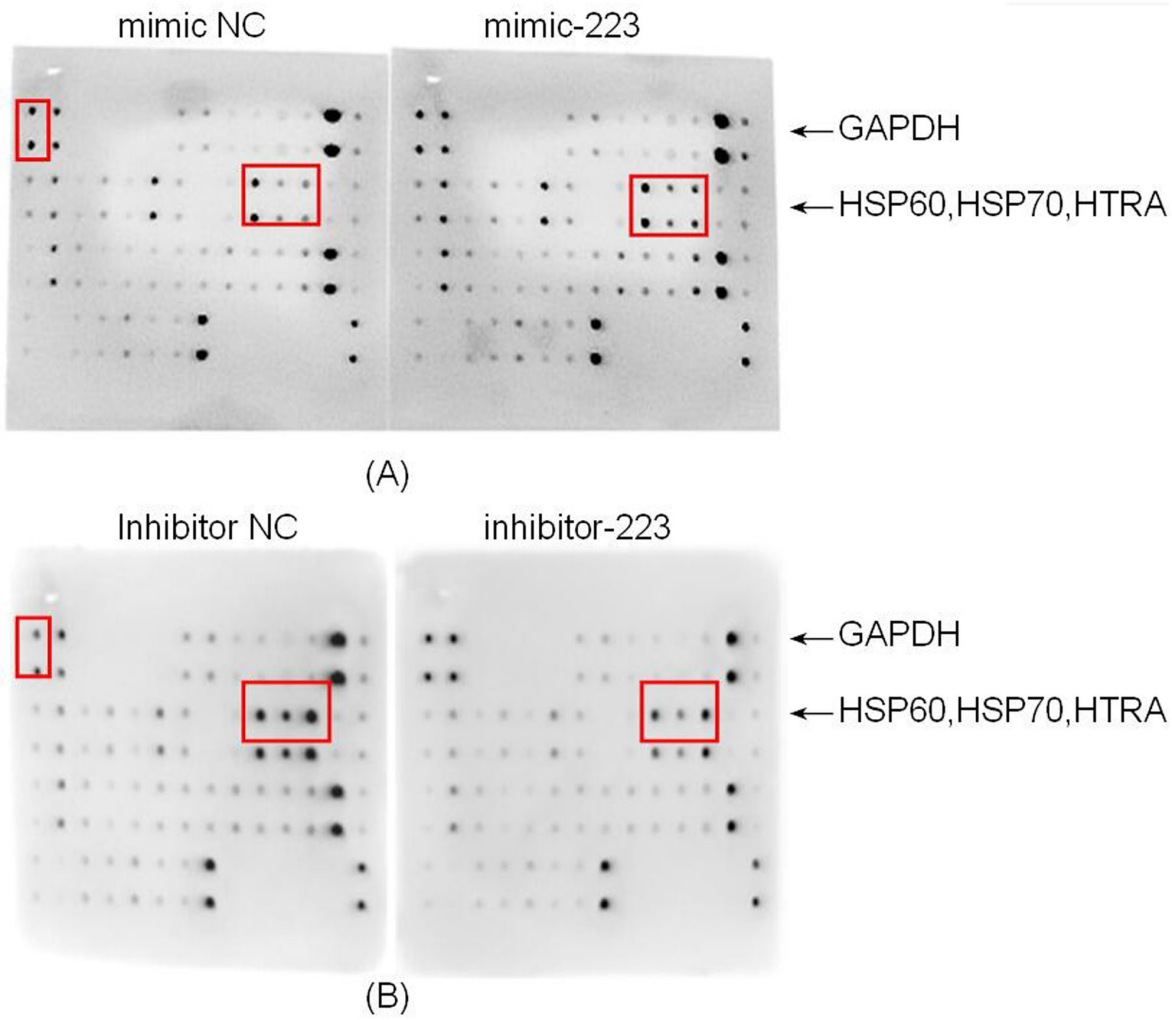
The probable downstream protein regulated by transcription factor FOXO1. (**A**,**B**) HSP60, HSP70, and HTRA were significantly increased in the mimic group and decreased in the inhibitor group compared to the control, indicating that the potential downstream molecules are HSP60, HSP70, and HTRA.

FOXO1 serves as a transcription factor and activates downstream apoptosis genes. Thus, we assessed downstream survival and apoptotic genes by using Human Apoptosis Antibody Array-Membrane (Abcam, ab134001). Among 41 proteins, HSP60, HSP70, and HTRA were found to be significantly increased in the mimic group (Fig. 6A) and decreased in the inhibitor group (Fig. 6B) compared to the control, indicating that HSP60, HSP70, and HTRA are potential downstream molecules. Supplemental files provide the quantification of the data in Fig. 6 using ImageJ (Table S2). Original figures are also provided (Fig. S6).

## Discussion

Early identification of sepsis-related mortality is vital to initiate rapid and appropriate therapeutic interventions. In recent years, researchers have focused on finding an ideal biomarker for the early diagnosis and prognosis of sepsis. Our previous study showed that some serum miRNAs can be used as biomarkers due to their predictive value in sepsis^22^. However, no studies have addressed the function of these miRNAs in immune cells or further investigated how they regulate downstream molecules in sepsis. Thus, to examine the potential role of miR-223 in target cells and not just for its diagnostic or prognostic value, we first evaluated expression of miR-223 in blood cells. Interestingly, we found that miR-223 was increased significantly in the septic group but decreased in the septic shock and nonsurvivor groups. We further investigated the potential role of miR-223 in immune cells.

According to TargetScan, FOXO1 is predicted to be a target gene of miR-223. Our prior research demonstrated that this protein is differentially expressed in normal controls and patients with sepsis, severe sepsis, and septic shock^15^. Previous studies^17^ have shown that the unphosphorylated form of FOXO1 is active and contributes to apoptosis and cell cycle dysfunction. In a rat model, lipopolysaccharide infusion induces an increase in AKT and FOXO1 gene transcription levels and a decrease in AKT and FOXO1 protein phosphorylation^23^. Our research first proved the vital role of miR-223 in cell survival and proliferation via regulation of FOXO1 in sepsis, in accordance with the function of FOXO1. Clinically, we found that miR-223 correlated negatively with SOFA scores and was significantly lower in nonsurviving patients with sepsis. Our work illustrates the mechanism relevant to clinical research.

FOXO1 functions as a transcription factor and activates downstream apoptosis genes (such as FasL, Trail)^16^. Thus, we examined downstream survival and apoptotic genes, including HSP60, HSP70, and HTRA, which regulate apoptosis as well as cell survival. These factors exhibit both anti-apoptotic^24^ and pro-apoptotic^25^ functions depending on the cell type and condition. Release of mitochondrial Hsp60 into the cytoplasm of intact Jurkat cells has been shown to accelerate caspase activation and promote apoptosis^25^. Interestingly, HTRA is associated with infectious diseases. Recent research shows that extracellular HTRAs are able to cleave cell-to-cell junction factors and promote bacterial colonization and invasion of host tissues^26^. This may be a new research target for sepsis.

In the course of sepsis, immune cells release a variety of cytokines during inflammation. This may result in the apoptosis of immune cells^27^. Because of the key role of the immune system in sepsis, immunomodulation represents an attractive target for adjunctive therapy. Until recently, clinical trials investigating immunomodulatory drugs have generally focused on suppressing the immune system based on the assumption that an overwhelming inflammatory response is the primary cause of death. Unfortunately, these trials failed to improve sepsis outcomes^1,2^. The apoptosis of immune cells is also regarded as a vital mechanism of immunosuppression^28^. Our research may provide new immunomodulatory targets for sepsis treatment. Overall, targeting miRNAs may be a new strategy for the treatment of septic patients.

## Supplementary information

Supplementary Information.

## References

[1] Singer M, Deutschman CS, Seymour CW, Shankar-Hari M, Annane D, Bauer M, Bellomo R, Bernard GR, Chiche JD, Coopersmith CM, Hotchkiss RS, Levy MM, Marshall JC, Martin GS, Opal SM, Rubenfeld GD, van der Poll T, Vincent JL, Angus DC (2016). The third international consensus definitions for sepsis and septic shock (Sepsis-3). JAMA.

[2] Iskander KN (2013). Sepsis: multiple abnormalities, hesterogeneous responses, and evolving understanding. Physiol. Rev..

[3] Boomer JS, To K, Chang KC, Takasu O, Osborne DF, Wal-ton AH (2011). Immunosuppression in patients who die of sepsis and multiple organ failure. JAMA.

[4] Hotchkiss RS, Swanson PE, Freeman BD (1999). Apoptotic cell death in patients with sepsis, shock, and multiple organ dysfunction. Crit. Care Med..

[5] Leliefeld PH, Wessels CM, Leenen LP (2016). The role of neutrophils in immune dysfunction during severe inflammation. Crit. Care.

[6] Squier MK, Sehnert AJ, Cohen JJ (1995). Apoptosis in leukocytes. J. Leukocyte Biol..

[7] Chung CS, Xu YX, Wang W, Chaudry IH, Ayala A (1998). Is Fas ligand or endotoxin responsible for mucosal lymphocyte apoptosis in sepsis?. Arch. Surg..

[8] Huttunen R, Syrjänen J, Vuento R, Laine J, Hurme M, Aittoniemi J (2012). Apoptosis markers soluble Fas (sFas), Fas Lig-and (FasL) and sFas/FasL ratio in patients with bacteremia: a prospective cohort study. J Infection.

[9] Lagos-Quintana M, Rauhut R, Lendeckel W, Tuschl T (2001). Identification of novel genes coding for small expressed rnas. Science.

[10] van der Heide V, Mohnle P, Rink J, Briegel J, Kreth S (2016). Down-regulation of microRNA-31 in CD4+ T cells contributes to immunosuppression in human sepsis by promoting TH2 skewing. Anesthesiology.

[11] Lodise TP, Mc Kinnon PS, Swiderski L, Rybak MJ (2003). Outcomes analysis of delayed antibiotic treatment for hospital-acquired Staphylococcus aureus bacteremia. Clin. Infect. Dis..

[12] Zhang X, Liu D, Liu YN, Wang R, Xie LX (2015). The accuracy of presepsin (sCD14-ST) for the diagnosis of sepsis in adults: a meta-analysis. Crit. Care.

[13] Liu D, Su LX, Han GC, Yan P, Xie LX (2015). Prognostic value of procalcitonin in adult patients with sepsis: a systematic review and meta-analysis. PLoS ONE.

[14] Wang H, Zhang P, Chen W, Feng D, Xie L (2012). Serum microRNA signatures identified by solexasequencing predict sepsis patients’ mortality: aprospective observational study. PLoS ONE.

[15] Wang HJ (2014). Identification of four novel serum protein biomarkers in sepsis patients encoded by target genes of sepsis-related miRNAs. Clin. Sci. (Lond).

[16] Benz F, Roy S, Trautwein C, Roderburg C, Luedde T (2016). Circulating microRNAs as biomarkers for sepsis. Int. J. Mol. Sci..

[17] Greer EL, Brunet A (2005). Foxo transcription factors at the interface between longevity and tumor suppression. Oncogene.

[18] Dragomir MP (2019). The non-coding RNome after splenectomy. Cell. Mol. Med..

[19] Aziz F (2016). The emerging role of miR-223 as novel potential diagnostic and therapeutic target for inflammatory disorders. Cell Immunol..

[20] Rhodes A, Evans LE, Alhazzani W (2017). Surviving sepsis campaign: international guidelines for the management of sepsis and septic shock: 2016. Crit. Care Med..

[21] Singer M, Deutschman CS, Seymour CW (2016). The third international consensus definitions for sepsis and septic shock (sepsis-3). JAMA.

[22] Wang H, Zhang P, Xie L (2012). Evidence for serum miR-15a and miR-16 levels as biomarkers that distinguish sepsis from systemic inflammatory response in human subjects. Clin. Chem. Lab. Med..

[23] Crossland H, Constantin-Teodosiu D, Gardiner SM (2008). A potential role for Akt/FOXO signalling in both protein loss and the impairment of muscle carbohydrate oxidation during sepsis in rodent skeletal muscle. J. Physiol..

[24] Shan YX, Liu TJ, Su HF, Samsamshariat A, Mestril R, Wang PH (2003). Hsp10 and Hsp60 modulate Bcl-2 family and mitochondria apoptosis signaling induced by doxorubicin in cardiac muscle cells. J. Mol. Cell Cardiol..

[25] Samali A, Cai J, Zhivotovsky B, Jones DP, Orrenius S (1999). Presence of a pre-apoptotic complex of pro-caspase-3, Hsp60 and Hsp10 in the mitochondrial fraction of jurkat cells. EMBO J.

[26] Backert S, Bernegger S, Skórko-Glonek J, Wessler S (2018). Extracellular HtrA serine proteases: an emerging new strategy in bacterial pathogenesis. Cell Microbiol..

[27] Arens C, Bajwa SA, Koch C (2016). Sepsis-induced long-term immune paralysis-results of a descriptive, explorative study. Crit. Care.

[28] Hamers L, Kox M, Pickkers P (2015). Sepsis-induced immunoparalysis: mechanisms, markers, and treatment options. Minerva Anestesiol..

