# Supplementary material for: The protective role of miR-223 in sepsis-induced mortality
Source: Sci Rep. 2020 Oct 19;10:17691. doi: 10.1038/s41598-020-74965-2 (PMC7572423; doi:10.1038/s41598-020-74965-2)
Supplement: Supplementary file 1 — Supplementary Information. [file 41598_2020_74965_MOESM1_ESM.doc]

**The protective role of miR-223 in sepsis-induced mortality.**

Dan Liu^1#^, Zhiding Wang^2#^, Huijuan Wang^1^, Feifei Ren^1^, Yanqin Li^1^, Sifan Zou^1^, Jianqiao Xu^1^, Lixin Xie^1*^

#Dan Liu and Zhiding Wang contributed equally to this work.

^1^ College of Pulmonary and Critical Care Medicine, Chinese PLA General Hospital, 28 Fuxing Road, Beijing, 100853, China

^2^ Department of Hematology and Oncology, International Cancer Center, Shenzhen University General Hospital, Shenzhen University Health Science Center, Shenzhen 518000, China

^*^Corresponding author

Email: dianebobo@126.com


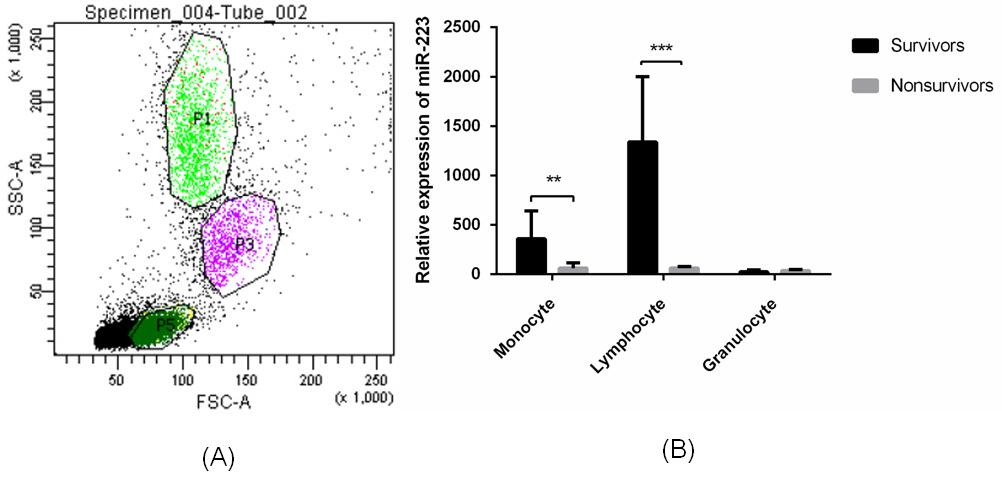


**Figure S1. Expression of miR-223 in various types of blood cells.** (A) Fresh peripheral blood cells were sorted into monocytes, lymphocytes, and neutrophils using FCM. Monocytes, lymphocytes, and neutrophils were collected, and qRT-PCR was used to detect the expression of miR-223 in these cells. (B) miR-223 was significantly higher in the lymphocytes in survivors compared with that of non-survivors.





**Figure S2. Results of transfection.** miR-223 was overexpressed in the Jurkat T cell lines in the mimic group and reduced in the inhibitor group compared to the NC group. **P* < 0.001.


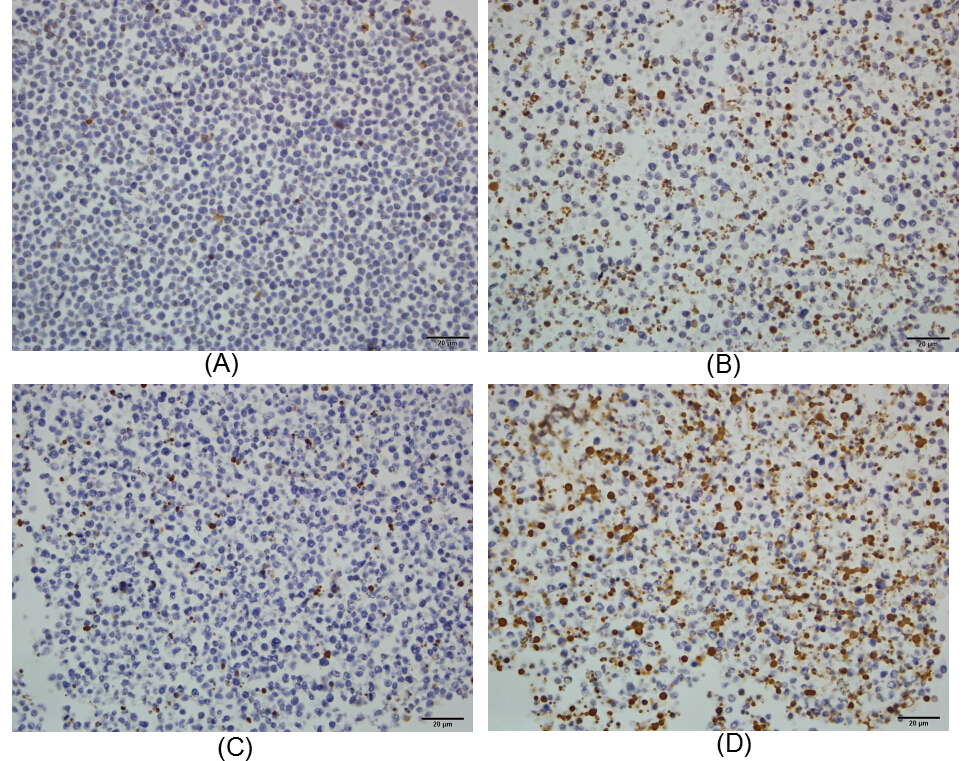


**Figure S3. Fas mediated-apoptosis in human Jurkat T cells induced by miR-223 treatment compared to that of the control as determined by TUNEL assays.** TUNEL-positive nuclei due to DNA fragmentation are shown as brown condensed spots. (A) Without treatment as a negative control; (B) Negative control treated with CH-11; (C) Jurkat T cells treated with CH-11 and miR-223 mimic; (D) Jurkat T cells treated with CH-11 and miR-223 inhibitor. Apoptosis was significantly decreased in the miR-223 mimic group compared with the cells only treated with CH-11. The miR-223 inhibitor group showed the reverse trend.


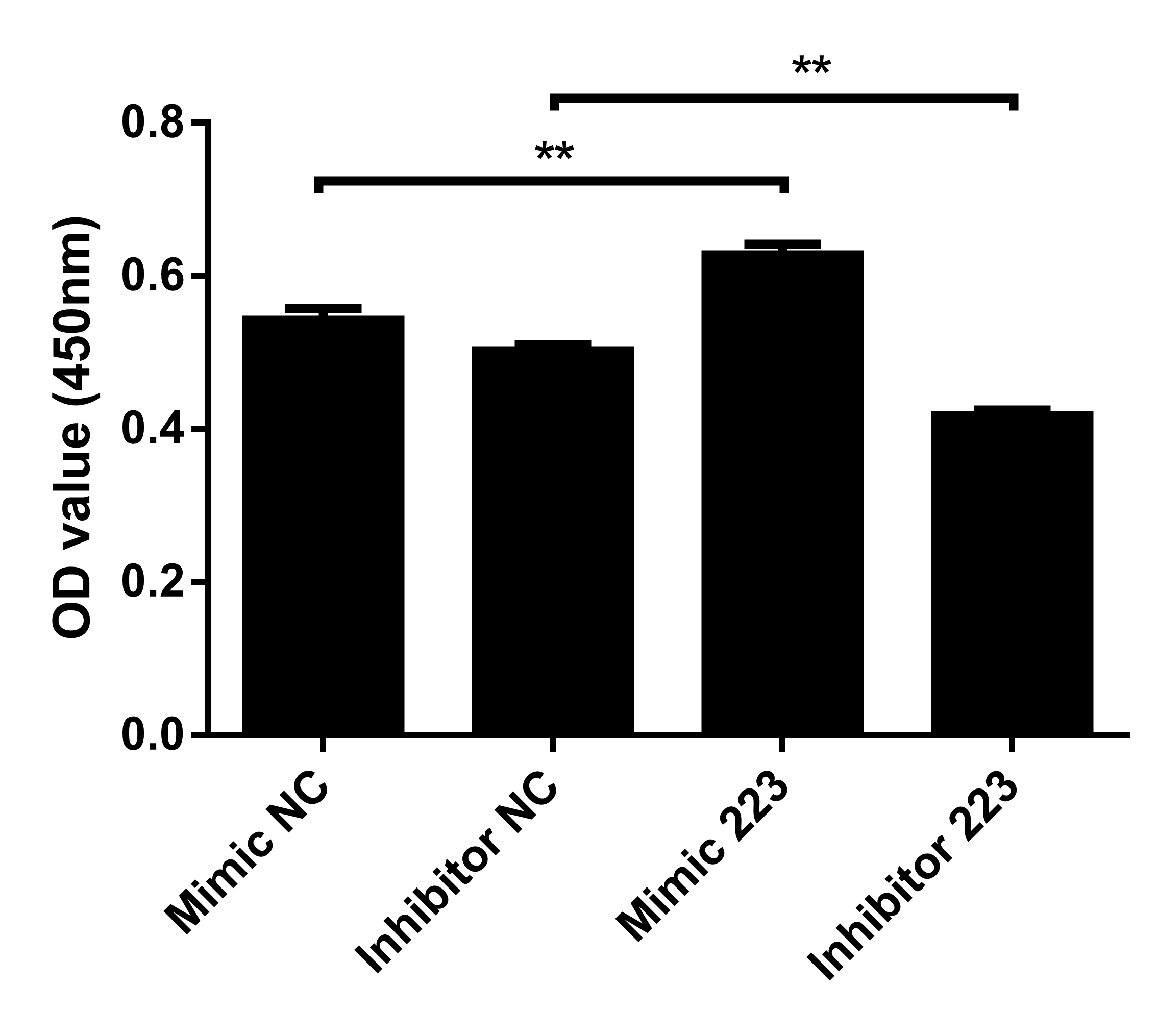


**Figure S4. miR-223 associated with cell proliferation.** In the WST-1 assay, the larger OD value indicated more active cell proliferation. The OD value of the miR-223 mimic group was significantly higher than that of the control, whereas the inhibitor group showed the reverse trend.


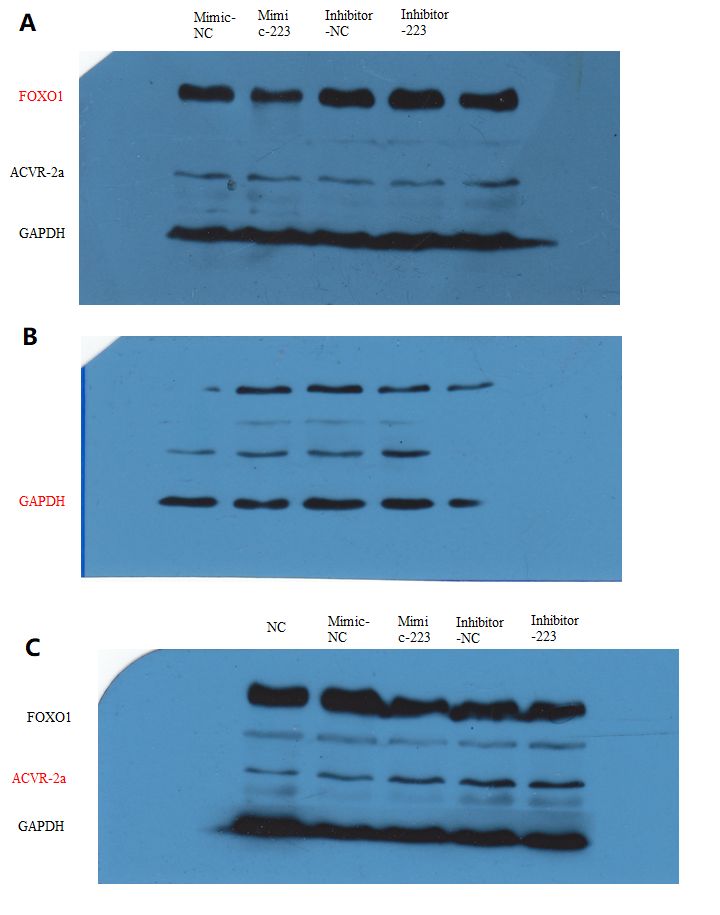


**Figure S5. The original figure of Figure 5 in manuscript**.


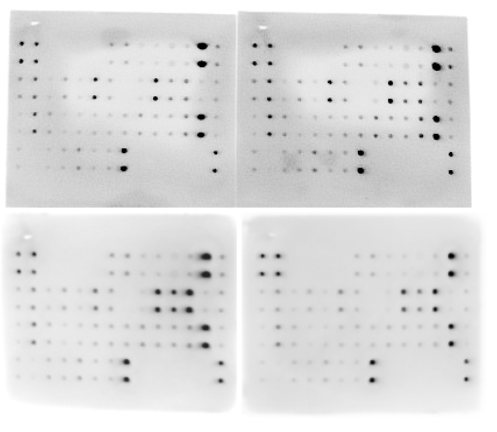


**Figure S6. The original figure of Figure 6 in manuscript**

|  | mimic NC | mimic223 | inhibitor NC | inhibitor 223 |
| --- | --- | --- | --- | --- |
| FOXO1 | 153265 | 143185 | 159634 | 164450 |
| ACVR2A | 143967 | 156210 | 137507 | 154400 |
| GAPDH | 153132 | 148253 | 155037 | 156387 |
| FOXO1/GAPDH | 1.001 | 0.966 | 1.030 | 1.052 |
| AVCR2A/GAPDH | 0.940 | 1.054 | 0.887 | 0.987 |

**S1 Table. Quantified the data of Figure 5 in manuscript by using ImageJ**

| Sample | Gene | Value | | Ratio | | |
| --- | --- | --- | --- | --- | --- | --- |
| mimic NC | PC* | 1831 | 1680 |  |  |  |
|  |  | 2241 | 2267 |  |  |  |
|  | Caspase-3 | 5905 | 5454 | Caspase-3/PC | 2.947 | 2.722 |
|  | HTRA | 1583 | 1645 | HTRA/PC | 0.790 | 0.821 |
|  | HSP70 | 1013 | 1180 | HSP70/PC | 0.505 | 0.589 |
|  | HSP60 | 3589 | 3775 | HSP60/PC | 1.791 | 1.884 |
| mimic 223 | PC | 2616 | 2188 |  |  |  |
|  |  | 2325 | 2315 |  |  |  |
|  | Caspase-3 | 6164 | 7460 | Caspase-3/PC | 2.611 | 3.160 |
|  | HTRA | 2418 | 2636 | HTRA/PC | 1.024 | 1.116 |
|  | HSP70 | 1679 | 1666 | HSP70/PC | 0.711 | 0.706 |
|  | HSP60 | 4420 | 4489 | HSP60/PC | 1.872 | 1.901 |
| inhibitor NC | PC | 2129 | 1124 |  |  |  |
|  |  | 1724 | 1726 |  |  |  |
|  | Caspase-3 | 6886 | 6922 | Caspase-3/PC | 4.111 | 4.133 |
|  | HTRA | 6242 | 6273 | HTRA/PC | 3.726 | 3.745 |
|  | HSP70 | 3699 | 3755 | HSP70/PC | 2.208 | 2.242 |
|  | HSP60 | 4233 | 4331 | HSP60/PC | 2.527 | 2.586 |
| inhibitor 223 | PC | 2433 | 2472 |  |  |  |
|  |  | 1798 | 1872 |  |  |  |
|  | Caspase-3 | 4566 | 4113 | Caspase-3/PC | 2.130 | 1.919 |
|  | HTRA | 3450 | 3384 | HTRA/PC | 1.617 | 1.586 |
|  | HSP70 | 1441 | 1312 | HSP70/PC | 0.675 | 0.615 |
|  | HSP60 | 2713 | 1993 | HSP60/PC | 1.271 | 0.934 |

*PC (Positive Control)

**S2 Table. Quantified the data of Figure 6 in revised manuscript by using ImageJ**

| Variables | Survivors(n=5) | Nonsurvivors(n=7) |
| --- | --- | --- |
| Gender(n)(male/female) | 2/3 | 4/3 |
| Age(years) | 51.80±23.35 | 82.57±10.690 |
| APACHEII score | 14.000±6.364 | 19.14±3.934 |
| SOFA score | 3.000±2.000 | 7.000±3.830 |
| CRP(mg/dl) | 12.132±9.745 | 12.132±9.745 |
| PCT(ng/ml) | 12.556±8.955 | 6.571±5.020 |
| WBC(×109/l) | 14.332±5.099 | 11.759±6.194 |
| miR-223(cycles) | 57.134±25.553 | 2.400±1.541 |

**S3 Table. Characteristics and hematological parameters of the 12 sepsis patients**
